# Reference Genes for Expression Analyses by RT-qPCR in *Hyblaea puera* (Lepidoptera: Hyblaeidae)

**DOI:** 10.3390/insects17060639

**Published:** 2026-06-17

**Authors:** Xinan Li, Ruiyang Qin, Wen Zhang, Fengfan Wang, Ganyu Zhu, Xiaopeng Wang, Hengyu Zhang, Menghui Liu, Liangjian Qu, Hao Yu

**Affiliations:** 1 Henan Engineering Technology Research Center for Major Invasive Alien Species Prevention and Control, School of Plant Protection and Environment, Henan Institute of Science and Technology, Xinxiang 453003, China; lixinan2019@126.com (X.L.); 15290364144@163.com (R.Q.); 15090369547@126.com (W.Z.); wangfengfanwff@163.com (F.W.); 15515255356@163.com (G.Z.); wangxiaopeng202604@163.com (X.W.); 19949301908@163.com (H.Z.); m15936038896@163.com (M.L.); 2 The Key Laboratory of Forest Protection of National Forestry and Grassland Administration, Ecology and Nature Conservation Institute, Chinese Academy of Forestry, Beijing 100091, China

**Keywords:** *Hyblaea puera*, RT-qPCR, reference gene, RefFinder, normalization

## Abstract

*Hyblaea puera* is a major pest of teak and mangroves. Reliable RT-qPCR normalization requires stable reference genes, none of which have been validated in *H*. *puera*. In this study, we assessed the expression stability of ten candidate reference genes under different experimental conditions. Stability was evaluated using the ΔCt method, BestKeeper, NormFinder, and geNorm, and a comprehensive stability ranking was generated using the RefFinder online tool. Our results indicated that amplification efficiencies ranged from 91.67% to 100.82%, with R^2^ values exceeding 0.9901. The optimal reference gene combinations varied by condition: *Ribosomal Protein L27* (*RPL27*) and *Ribosomal Protein L10* (*RPL10*) for temperature treatments; *Actin* and *RPL10* for larval instars; *Ribosomal protein S5* (*RPS5*) and *Elongation factor-1α* (*EF-1a*) for adult sexes; *RPL10* and *EF-1a* for tall developmental stages; *RPL10* and *RPS5* for tissues; as well as *EF-1α* and *Actin* for all combined conditions. Finally, the expression profiles of target gene *Lethal* were evaluated, and the outcomes further confirm the importance of selecting fitting reference genes for normalization of qRT-PCR data. These results provide the evaluated reference gene sets for *H*. *puera*, facilitating more accurate RT-qPCR normalization in future molecular studies of host plant adaptation (teak vs. mangroves), temperature tolerance, and larval development in this pest.

## Simple Summary

To achieve accurate gene expression analysis using real-time quantitative reverse transcription polymerase chain reaction (RT-qPCR), the expression stability of candidate reference genes must be validated under specific experimental conditions before they can be used to normalize the expression levels of target genes. The teak defoliator, *Hyblaea puera* (Cramer), is a major invasive pest native to Southeast Asia and is one of the most destructive defoliators in teak (*Tectona grandis*) plantations. In recent years, severe damage caused by this pest to mangrove plants has been reported multiple times, posing a serious threat to the security of mangrove ecosystems in coastal regions. However, research on this pest remains very limited. In this study, we evaluated the suitability of ten commonly used reference genes for RT-qPCR data normalization in *H*. *puera* under different experimental conditions. Our evaluated reference gene sets will enable future investigations into the molecular mechanisms underlying its host shifts, developmental regulation, and stress responses, which are critical for understanding its outbreak dynamics and developing effective control strategies. These findings provide a foundation for establishing a more rigorous RT-qPCR normalization framework in this economically and ecologically significant pest.

## 1. Introduction

The teak defoliator, *Hyblaea puera* (Cramer), is an invasive pest belonging to the order Lepidoptera and the family Hyblaeidae; native to Southeast Asia, it ranks among the most destructive foliar pests in teak (*Tectona grandis*) plantations [1]. Following the global introduction of teak, the distribution of *H*. *puera* has expanded to Latin America, where it poses a significant threat to local teak cultivation. This pest exhibits a broad host range and is capable of feeding on various plant species beyond teak [2]. In recent years, multiple studies have reported this pest infesting mangrove plants (*Avicennia*), constituting a serious threat to mangrove ecosystem security. The defoliation it causes has led to severe degradation of mangrove ecosystems [3]. Documented outbreaks of this pest in mangroves have occurred in regions including India [4], Brazil, and coastal areas of Hainan and Guangxi, China [5,6]. Despite the increasing number of reports documenting its damage to mangroves, research on this pest remains notably limited.

Reverse transcriptase-quantitative polymerase chain reaction (RT-qPCR) is regarded as the optimal approach for gene expression and transcriptome analysis due to its high sensitivity, reproducibility, and specificity, and can be effectively performed on high-throughput platforms [7,8]. Reliable RT-qPCR results depend on accurate transcript normalization [9]. Numerous factors can significantly influence threshold cycle (Ct) values, including RNA quality and quantity, variations in transcriptional efficiency, primer characteristics, and PCR conditions [10,11]. In most analytical approaches, the application of reference genes helps to eliminate discrepancies arising from differences in sample purity and concentration, thereby facilitating valid comparisons of target gene expression across samples [12]. Therefore, the identification of appropriate and stable reference genes for use as internal controls is critical for the normalization of expression levels [13,14].

In gene expression normalization analyses, housekeeping genes involved in fundamental and ubiquitous cellular functions are frequently employed as reference genes [15,16], such as those encoding *Actin*, *elongation factor 1α (EF-1a)*, *glyceraldehyde-3-phosphate dehydrogenase* (*GAPDH*), *ribosomal proteins*, *β-tubulin*, and *glutathione S-transferase*. An ideal reference gene should exhibit stable expression levels and remain unaffected by experimental conditions or environmental variations [17,18]. Failure to carefully assess the suitability of these genes under specific experimental conditions may lead to inaccuracies or inconsistencies in RT-qPCR data [19]. There is no universal reference gene applicable to all gene expression analyses [20]. Consequently, it is necessary to systematically evaluate the suitability of candidate reference genes under specific experimental conditions.

To date, several methods and tools have been developed to assess reference gene stability, including the NormFinder [21], ΔCt method [22], geNorm [23], BestKeeper [24], and the web-based analytical tool RefFinder [25]. Given the importance of reference genes in RT-qPCR analysis, previous studies have validated reference gene sets for various lepidopteran pests, including *Spodoptera exigua* [26], *Chilo Sacchariphagus* [27], *Spodoptera frugiperda* [28], *Lymantria dispar* [29], *Sesamia inferens* [30], *Chilo partellus* [15], and *Helicoverpa armigera* [31]. However, the expression stability of potential housekeeping genes in *H*. *puera* remains unverified.

In this study, we evaluated the suitability of ten commonly used reference genes-*Actin*, *GAPDH*, *Arginine Kinase* (*AK*), *Ribosomal Protein L10* (*RPL10*), *Ribosomal Protein L13* (*RPL13*), *Beta-tubulin* (*Beta*), *Ribosomal Protein L27* (*RPL27*), *EF-1a*, *Ribosomal protein S5* (*RPS5*), and *28S ribosomal RNA* (*28S*)-for normalizing RT-qPCR data in *H. puera*. The effects of varying temperature treatments, larval instars, adult sexes, total developmental stages, and tissue types on the expression of these candidate genes were systematically assessed. In addition, the expression patterns of the longevity signaling pathway gene *Lethal* under different experimental conditions mentioned above were evaluated to verify the results. Our findings provide a technical resource for more rigorous RT-qPCR normalization and functional gene studies in *H. puera*.

## 2. Materials and Methods

### 2.1. Insects

Larvae of *H. puera* were collected in 2024 from a Mangrove Nature Reserve located in Guangxi, China (coordinates: 21°28′24″ N, 109°43′57″ E). Rearing was conducted indoors using an artificial diet in a climate-controlled chamber set at 26 ± 2 °C, 65% ± 5% humidity, and a 16-h light/8-h dark cycle. Newly emerged adults were transferred to clean cages and fed a 10% honey solution.

### 2.2. Treatments

Larval instars. Individuals at different larval stages were collected as independent samples, including 10 first-instar larvae, 10 s-instar larvae, 5 third-instar larvae, 3 fourth-instar larvae, 3 fifth-instar larvae, to analyze gene expression across different larval developmental stages.

Adult sexes. Three-day-old healthy male and female adults were collected separately, with three adults of the same sex pooled into one sample.

Total developmental stage. Samples from the different larval stages and adult sexes mentioned above, together with three pupae as one sample, were used to analyze gene expression across the total developmental stage.

Body part. Fifth-instar larvae were dissected to obtain head, thorax, and abdomen tissues. The whole abdomen was dissected and briefly rinsed in sterile water to remove surface debris and loose gut contents. Tissues from five larvae were pooled into one sample for each tissue type.

Temperature-induced stress. Fourth-instar larvae were randomly assigned to five temperature treatments (15 °C, 20 °C, 25 °C, 30 °C, and 35 °C) in climate-controlled incubators for 24 h. Following exposure, three larvae were pooled to form a single sample. No mortality was recorded across all temperature groups during the experiment.

There is a total of 16 types of samples as described above, with three biological replicates for each sample, amounting to 48 samples in total. All collected samples were immediately flash-frozen in liquid nitrogen and stored at −80 °C until further use.

### 2.3. Total RNA Extraction and cDNA Synthesis

Total RNA was isolated with Trizol reagent according to the TRNzol Universal Reagent protocol provided by the manufacturer (Tiangen, Beijing, China). The A260/A280 absorbance ratios of the samples fell between 1.9 and 2.1, reflecting high RNA purity. First-strand cDNA was synthesized from 1 μg of RNA template with the FastKing gDNA Dispelling RT SuperMix (Tiangen, Beijing, China) following the manufacturer’s recommended protocol. The synthesized cDNA was stored at −20 °C.

### 2.4. Primer Design and RT-qPCR

Ten commonly used reference genes (*Actin*, *GAPDH*, *AK*, *RPL10*, *RPL13*, *RPL27*, *EF-1a*, *Beta*, *RPS5*, and *28S*) were selected as candidate genes from the transcriptome data of fifth-instar *H*. *puera* larvae (unpublished). All primers were designed with Primer Premier 5.0 (PREMIER Biosoft International, Palo Alto, CA, USA). Verification of amplification specificity for each primer pair was performed using 1% agarose gel electrophoresis and melting curve analysis. Detailed primer sequences and related information for RT-qPCR are listed in Table 1.

A CFX Connect Real-Time System (Bio-Rad, Hercules, CA, USA) was employed for RT-qPCR, with the Talent qPCR PreMix (SYBR Green, Tiangen, Beijing, China). The amplification mixture (total volume 25 µL) was composed of 12.5 µL TB Green Premix Ex Taq II (Tli RNaseH Plus), 9.5 µL nuclease-free water, 1 µL each of forward and reverse primers (10 µM), and 1 µL cDNA template. Cycling conditions were set as: 95 °C for 2 min, then 40 cycles of 95 °C for 5 s and 60 °C for 15 s. Standard curves were established via a linear regression model using 10-fold serial dilutions of cDNA. Three biological and three technical replicates were performed for each analysis. The nucleotide sequences of the ten candidate reference gene PCR products from *H. puera* are shown in Supplementary Materials Text S1.

### 2.5. Evaluation of Reference Gene Expression Stability

The stability and comprehensive ranking of the ten candidate reference genes under different experimental conditions was assessed and determined using a web-based tool RefFinder (https://blooge.cn/RefFinder, accessed on 16 January 2026), which integrates the four algorithms mentioned (geNorm, NormFinder, BestKeeper, and the ΔCt method) [32]. The optimal number of reference genes for accurate normalization was determined by calculating pairwise variation (V*n*/V*n*+1) between sequential normalization factors (NF) using geNorm. A V*n*/V*n*+1 below 0.15 indicates that adding another reference gene yields no significant improvement.

### 2.6. Validation of Recommended Reference Genes

To assess the reliability of the experimental results, the transcriptional levels of the longevity signaling pathway gene *Lethal* in *H. puera* were analyzed under different experimental conditions. The *Lethal* gene was obtained from our transcriptome sequencing data, and its sequence is provided in Supplementary Materials Text S2. The primer sequences used were F: CCAGACCGAAGTCCTGTTCC and R: CAATTGGTGCGAGAGCACTG. Three biological replicates were performed for each condition. Relative expression levels were calculated using the 2^−ΔΔCt^ method. To compare the expression levels of *Lethal* normalized by different reference genes, statistical analyses were performed using one-way ANOVA in IBM SPSS Statistics v31. Specifically, comparisons were made among the expression levels normalized by the most stable reference gene, the recommended combination of reference genes, and the least stable reference gene.

## 3. Results

### 3.1. Amplification Efficiencies

**Figure 1:**
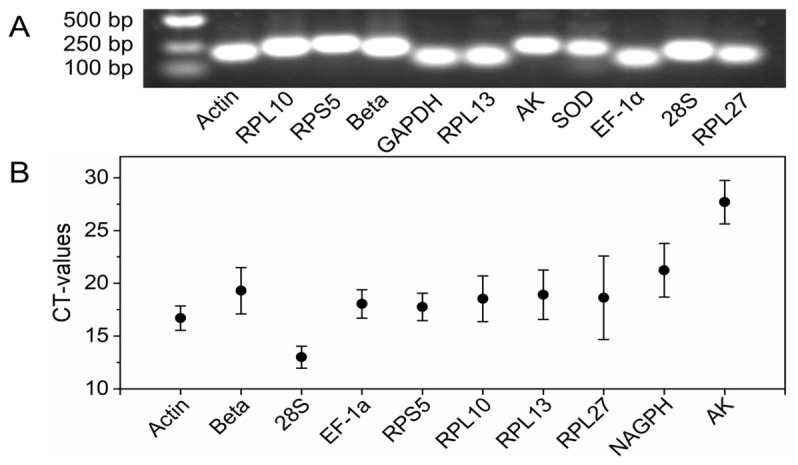
PCR product detection (**A**) and expression profiles (**B**) of ten candidate reference genes in *Hyblaea puera*. Black dots represent the average Ct values for all samples (*n* = 48). The bar indicates the standard deviation (SD).

RT-qPCR analysis revealed that all candidate reference genes were expressed in *H*. *puera*. PCR products for each gene were detected as single, clear amplification bands of the expected size on 1% agarose gels (Figure 1A). Amplification efficiencies were evaluated using five-point standard curves, with efficiency (E) values ranging from 91.67% to 100.82% and regression coefficients (R^2^) exceeding 0.9901 for all genes (Table 1). Furthermore, melting curve analysis exhibited a single specific peak for each gene without any primer-dimer peaks (Figure S1), further confirming the amplification specificity of each primer pair.

### 3.2. Expression Profiles of Candidate Reference Genes

The gene expression levels of the 10 candidate reference genes exhibited a wide range of Ct values across all experimental conditions. The Ct values ranged from 10.60 for the *28S* gene to 33.24 for the *AK* gene. The mean Ct value of the *28S* gene was 13.00, which was considerably lower than those of the other genes, indicating high expression of this gene under all conditions. The *AK* gene had a mean Ct value of 27.69, which was higher than those of the other genes. The remaining eight candidate reference genes displayed moderate expression levels. Specifically, the mean Ct values for the *Actin*, *Beta*, *EF-1a*, *RPS5*, *RPL10*, *RPL13*, *RPL27*, and *GAPDH* genes were 16.70, 19.30, 18.04, 17.76, 18.53, 18.91, 18.62, and 21.23, respectively (Figure 1B).

### 3.3. Stability of Candidate Reference Genes

**Figure 2:**
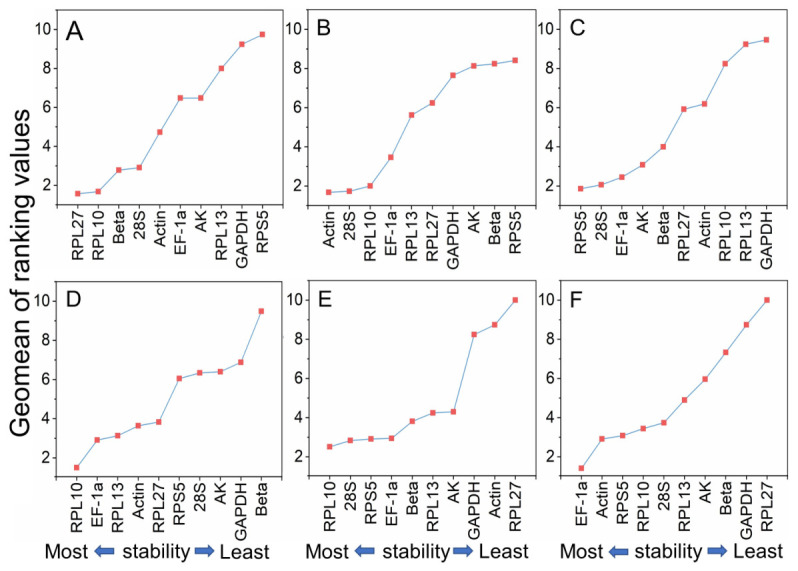
Ranking of candidate reference genes based on expression stability, as determined by RefFinder. A lower geomean of ranking values indicates higher expression stability. The stability was evaluated across various experimental conditions: (**A**) Temperature, (**B**) Larval instars, (**C**) Adult sexes, (**D**) Total developmental stages, (**E**) Tissue types, (**F**) All conditions.

**Figure 3:**
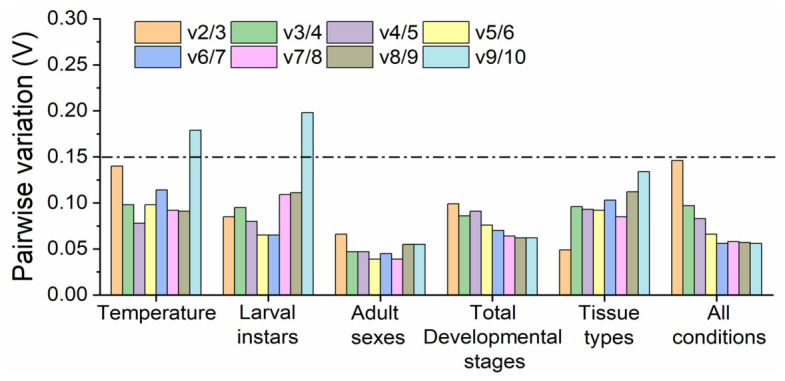
Determination of the optimal number of reference genes for accurate normalization using geNorm. The V*n*/*n*+1 value indicates the pairwise variation (Y axis) between two sequential normalization factors and determines the optimal number of reference genes required for an accurate data normalization. A value below 0.15 indicates that an additional reference gene will not significantly improve the normalization.

Temperature. Under different temperature treatments, the ΔCt method, NormFinder, and geNorm identified *RPL27* and *RPL10* as the most stable candidate reference genes, whereas BestKeeper ranked *Beta* and *28S* as the most stable. *GAPDH* and *RPS5* were consistently ranked as the least stable by all four methods (Table 2). According to the comprehensive ranking from RefFinder, the stability order from most to least stable was: *RPL27* > *RPL10* > *Beta* > *28S* > *Actin* > *EF-1α* > *AK* > *RPL13* > *GAPDH* > *RPS5* (Figure 2A). The pairwise variation value V2/3 calculated by geNorm was 0.148 (Figure 3), which is below the commonly accepted threshold of 0.15, indicating that two reference genes are sufficient for accurate normalization. Accordingly, *RPL27* and *RPL10* are recommended as the optimal reference genes for temperature treatment experiments (Table 3).

Larval instars. For different larval instars, the ΔCt method and NormFinder identified *RPL10* and *Actin* as the most stable candidate reference genes, whereas BestKeeper and geNorm ranked *28S* and *Actin* as the most stable. *AK* and *RPS5* were consistently identified as the least stable by the ΔCt method, NormFinder, and geNorm, while BestKeeper ranked *Beta* and *GAPDH* as the least stable (Table 2). According to the comprehensive ranking from RefFinder, the stability order from most to least stable was: *Actin* > *28S* > *RPL10* > *EF-1α* > *RPL13* > *RPL27* > *GAPDH* > *AK* > *Beta* > *RPS5* (Figure 2B). The pairwise variation value V2/3 calculated by geNorm was 0.085 (Figure 3), which is well below the commonly accepted threshold of 0.15, indicating that two reference genes are sufficient for accurate normalization. Consequently, *Actin* and *RPL10* are recommended as the optimal reference genes for studies involving different larval instars (Table 3).

Adult sexes. For adult females and males, the ΔCt method identified *28S* and *RPS5* as the most stable reference genes, while NormFinder ranked *AK* and *28S* as the most stable. BestKeeper and geNorm both determined *EF-1α* and *RPS5* as the most stable candidates. The ΔCt method, NormFinder, and geNorm consistently identified *RPL13* and *GAPDH* as the least stable, whereas BestKeeper ranked *RPL10* and *RPL13* as the least stable (Table 2). According to the comprehensive ranking from RefFinder, the stability order from most to least stable was: *RPS5* > *28S* > *EF-1α* > *AK* > *Beta* > *RPL27* > *Actin* > *RPL10* > *RPL13* > *GAPDH* (Figure 2C). The pairwise variation value V2/3 calculated by geNorm was 0.066 (Figure 3), which is well below the commonly accepted threshold of 0.15, indicating that two reference genes are sufficient for accurate normalization. Therefore, *RPS5* and *EF-1α* are recommended as the optimal reference genes for adult sexes studies (Table 3).

Total developmental stages. For overall different developmental stages, ΔCt and Normfinder analyses identified *RPL10* and *EF-1a* as the most stable candidate housekeeping genes, BestKeeper analysis determined *Actin* and *28S* as the most stable, while geNorm analysis identified *RPL10* and *RPL13* as the most stable. The ΔCt, Normfinder, and geNorm analyses identified *28S* and *Beta* as the least stable candidate housekeeping genes, whereas BestKeeper analysis identified *Beta* and *GAPDH* as the least stable (Table 2). According to the comprehensive RefFinder analysis, the ranking of candidate housekeeping genes from most stable to least stable across overall different developmental stages was: *RPL10* > *EF-1a* > *RPL13* > *Actin* > *RPL27* > *RPS5* > *28S* > *AK* > *GAPDH* > *Beta* (Figure 2D). The geNorm analysis showed a pairwise variation value (V2/3) of 0.099 (Figure 3). Therefore, *RPL10* and *EF-1a* are recommended as the optimal reference genes for overall different developmental stages (Table 3).

Tissue types. For total tissues (including head, thorax, and abdomen), ΔCt, BestKeeper, Normfinder, and geNorm analyses identified *RPL10* and *28S*, *EF-1a* and *28S*, *AK* and *RPL10*, as well as *Beta* and *RPS5* as the most stable candidate housekeeping genes, respectively. The ΔCt, Normfinder, and geNorm analyses identified *Actin* and *RPL27* as the least stable candidate housekeeping genes, whereas BestKeeper analysis identified *GAPDH* and *RPL27* as the least stable (Table 2). According to the comprehensive RefFinder analysis, the ranking of candidate housekeeping genes from most stable to least stable in total tissues was: *RPL10* > *28S* > *RPS5* > *EF-1a* > *Beta* > *RPL13* > *AK* > *GAPDH* > *Actin* > *RPL27* (Figure 2E). The geNorm analysis showed a pairwise variation value (V2/3) of 0.049 (Figure 3). Therefore, *RPL10* and *RPS5* are recommended as the optimal reference genes for total tissues (Table 3).

All conditions. For all experimental conditions, BestKeeper identified *28S* and *Actin* as the most stable, geNorm identified *EF-1a* and *RPS5* as the most stable, while ΔCt and Normfinder analyses determined *EF-1a* and *RPL10* as the most stable candidate housekeeping genes. The ΔCt, BestKeeper, and Normfinder analyses identified *GAPDH* and *RPL27* as the least stable candidate housekeeping genes, whereas geNorm analysis identified *Beta* and *RPL27* as the least stable (Table 2). According to the comprehensive RefFinder analysis, the ranking of candidate housekeeping genes from most stable to least stable across all experimental conditions was: *EF-1a* > *Actin* > *RPS5* > *RPL10* > *28S* > *RPL13* > *AK* > *Beta* > *GAPDH* > *RPL27* (Figure 2F). The geNorm analysis showed a pairwise variation value (V2/3) of 0.147 (Figure 3). Therefore, *EF-1a* and *Actin* are recommended as the optimal reference genes for all experimental conditions (Table 3).

### 3.4. Validation of Recommended Reference Genes

**Figure 4:**
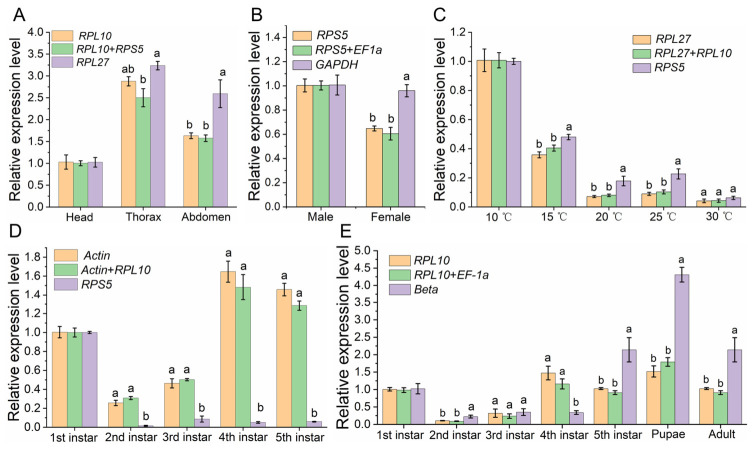
Relative expression levels of the target gene *Lethal* in (**A**) Tissue types, (**B**) Adult sexes, (**C**) Temperature, (**D**) Larval instars, (**E**) Total developmental stages were normalized by the most stable reference gene, the recommended the combination of the two most stable reference genes, and least stable reference gene. The data represents the mean values ± SE of three biological replicates. Different lowercase letters indicate significant differences at the 0.05 level.

To validate the stability of the recommended reference genes, the expression levels of the target gene *Lethal* under five experimental conditions were normalized using the most stable reference gene, the combination of the two most stable reference genes, and the least stable reference gene (Figure 4). For example, under different tissue conditions, with the expression level in the head used as a control, the expression level of *Lethal* in the thorax was higher than that in the other two tissues, regardless of whether *RPL10*, the combination of *RPL10* and *RPS5*, or *RPL27* was used for normalization. No significant difference was observed between the expression levels of *Lethal* in the thorax and abdomen normalized by *RPL10* and those normalized by the combination of *RPL10* and *RPS5*. However, both of these were significantly different from the expression levels normalized by *RPL27*. Similar results were obtained in most of the other experimental conditions, where no significant differences were observed between the expression levels normalized by the most stable reference gene and the combination of the two most stable reference genes, whereas significant differences were detected when the least stable reference gene was used for normalization.

## 4. Discussion

In this study, we systematically evaluated the expression stability of ten candidate reference genes in *H*. *puera* under various experimental conditions, including different temperatures, larval instars, adult sexes, total developmental stages, tissue types, and across all combined conditions. The aim was to identify the most suitable reference genes for accurate normalization of RT-qPCR data in *H. puera*. Our results demonstrated that no single reference gene exhibited universal stability across all experimental conditions, consistent with previous reports that the expression of housekeeping genes can vary significantly depending on the biological context [27,33,34]. Therefore, it is essential to validate reference gene stability under each specific experimental condition prior to their use in gene expression studies.

The amplification efficiencies of all ten candidate genes ranged from 91.67% to 100.82%, with R^2^ values exceeding 0.9901, indicating reliable and consistent amplification performance. Melting curve analysis further confirmed the specificity of each primer pair, as evidenced by single peaks without primer-dimer artifacts. These results ensure that the subsequent stability evaluations were based on specific and efficient amplification of the target genes [35,36]. The expression levels of the candidate reference genes varied widely across samples, with Ct values ranging from 10.60 (*28S*) to 33.24 (*AK*). The *28S* gene, a ribosomal RNA (rRNA) component, exhibited the highest overall expression, consistent with its role as a fundamental structural element of the ribosome [37]. Conversely, *AK* showed the lowest expression, reflecting the inherent variability in transcript abundance among commonly used reference genes [11,38]. This variability underscores the necessity of selecting stably expressed reference genes rather than relying on traditional housekeeping genes without prior validation [36,39].

Under different temperature treatments, the comprehensive RefFinder analysis ranked *RPL27* and *RPL10* as the optimal pair, indicating that two reference genes are sufficient for accurate normalization. Similarly, for different larval instars, *Actin* and *RPL10* were recommended as the optimal reference genes. These findings align with recent studies on Lepidoptera insects, which have consistently reported that ribosomal protein genes (e.g., *RPL10*, *RPL27*) and *EF-1α* exhibit high stability under temperature stress and across developmental stages. For instance, in the lepidopteran pest *Mythimna loreyi*, *RPL27* and *RPL10* were identified as the most suitable reference genes across developmental stages and temperature treatments [33]. Similarly, in *Phthorimaea operculella*, *RPL27* ranked as the optimal combination under temperature treatments [40]. The stability of ribosomal protein genes may be attributed to their constitutive role in maintaining protein synthesis, which is essential for cellular homeostasis even under environmental stress [41]. The developmental stage-specific expression stability of reference genes observed here is also supported by recent studies. In *S. frugiperda*, *RPL10* were recommended for developmental stages [28], while in *C. sacchariphagus*, the combination of *Actin* and *RPL7* was optimal for temperature treatments [27].

In adult females and males, *RPS5* and *28S* emerged as the most stable reference genes according to the comprehensive RefFinder ranking. Notably, *RPS5* was consistently identified as stable across multiple algorithms, suggesting its suitability for sex-specific expression studies. This finding aligns with recent studies in Lepidoptera, where ribosomal protein genes, including *RPS5* homologs, have demonstrated high stability across sexes [33]. However, *RPS5* was the least stable reference gene under different temperatures and across various larval stages. This discrepancy underscores the specificity of transcriptional regulation at both the species and gene levels. Kim et al. (2021) demonstrated that *RPS5* expression significantly differs across honeybee developmental stages [42], and is affected by external environmental factors [43]. For total developmental stages, *RPL10* and *EF-1a* were recommended as the optimal reference genes. This finding is consistent with reports in other insect species where *EF-1a* has been shown to be stably expressed during development [28,40]. Ribosomal protein genes such as *RPL10* are increasingly recognized as reliable references due to their constitutive roles in protein synthesis, essential for cellular homeostasis across developmental transitions [41]. For total tissue samples, *RPL10* and *28S* were identified as the most stable pair, indicating high stability across different tissue types. These results corroborate recent findings in *Scotogramma trifolii*, where *RPL10* was validated as a stable reference for adult tissues [44], and in *Athetis dissimilis*, where *28S* were ranked among the most stable genes for adult tissues [45]. Collectively, these findings underscore that ribosomal protein genes and *EF-1a* serve as reliable reference genes across diverse biological contexts in Lepidoptera.

The *28S* sequence encodes rRNA that is highly expressed in all biological cells, and its low Ct value observed in this study reflects the abundance of these transcripts (Figure 1). Although *28S* exhibited relatively high stability rankings across tissue types, adult sexes, and larval stages, the fundamental differences in rRNA and mRNA fractions between samples limit its use as a normalizer in RT-qPCR analyses [13,23]. Specifically, rRNA cannot reliably correct for sample-to-sample variation in mRNA quantity, as it may not represent mRNA levels [46]. Additionally, using 28S as a reference gene would require substantial sample dilution to bring rRNA within the detection range, which would concomitantly dilute target mRNA and risk over- or underestimating expression differences. Therefore, despite its computational stability rankings, we recommend excluding 28S as a reference gene in *H. puera* RT-qPCR studies.

When considering all experimental conditions collectively, *EF-1a* and *Actin* were identified as the most stable reference genes. This suggests that even across a wide range of biological conditions, these two genes maintain relatively stable expression and may serve as reliable internal controls for comprehensive expression studies in *H*. *puera*. Interestingly, some commonly used reference genes such as *GAPDH* and *Beta* were frequently ranked among the least stable across various conditions, consistent with previous observations that these genes can be differentially regulated in response to experimental treatments. Recent studies in Lepidoptera further support these findings. In *S. trifolii*, *Actin* was identified among the most stable reference genes for developmental stage normalization, while *GAPDH* showed condition-dependent stability [44]. Similarly, in *Pieris melete*, *EF1α* was recommended as an optimal reference gene under various experimental conditions [47]. In sand flies, *EF1-α* consistently ranked among the most stably expressed genes across developmental stages, tissues, and experimental treatments [48]. The instability of classical housekeeping genes such as *GAPDH* and *Beta* under specific conditions has also been documented in *S. frugiperda* [49] and *Perina nuda* [50], underscoring the necessity of validating reference genes prior to use. Together, these findings support *EF-1α* and *Actin* as reliable reference gene candidates for multi-condition expression studies in Lepidoptera.

To move beyond purely computational ranking and provide experimentally validated recommendations, the expression levels of the target gene Lethal were normalized using different reference gene sets under various experimental conditions. Our results demonstrated that, under most experimental conditions, the expression levels normalized using the most stable reference gene showed no significant difference from those normalized using the combination of the two most stable reference genes. In contrast, significant differences were observed when the least stable reference gene was used for normalization (*p* < 0.05) (Figure 4). This co-validation directly confirms that selecting inappropriate reference genes can lead to inaccurate quantification of target gene expression, underscoring the practical importance of the recommendations provided in this study.

## 5. Conclusions

In conclusion, this study provides a systematic evaluation of reference gene stability in *H. puera* under multiple experimental conditions. No single reference gene was universally suitable across all conditions, confirming that reference gene selection must be tailored to the specific experimental context. Based on our findings, we recommend *RPL27* and *RPL10* for temperature treatments, *Actin* and *RPL10* for different larval instars, *RPS5* and *EF-1a* for adult sex comparisons, *RPL10* and *EF-1a* for total developmental stages, *RPL10* and *RPS5* for tissue-specific studies, as well as *EF-1a* and *Actin* for experiments encompassing all conditions in this study. These recommendations serve as a technical resource for future functional gene expression studies in *H. puera*, providing a foundation for more rigorous RT-qPCR normalization in this understudied pest species.

## Figures and Tables

**Table 1:** Primer sequences and amplicon characteristics of the 10 reference genes.

Gene Symbol	Gene Name	Primer Sequences (5′ → 3′)	^a^ L (bp)	^b^ E (%)	^c^ R^2^	Slope
*Actin*	*Actin*	F: TACTCTTTCACCACCACCGC R: GGCAACGGAACCTCTCGTTA	181	93.31%	0.9982	−3.4932
*Beta*	*Beta-tubulin*	F: ATGTCTGGTGTGACGACGTG R: GCCATCATGTTCTTGGCGTC	206	95.29%	0.9901	−3.4402
*28S*	*28S ribosomal RNA*	F: GGCCGTTGGACGGTATATT R: GTGCCCGAAACGGAAACATC	178	94.04%	0.9953	−3.4735
*EF-1a*	*Elongation factor-1α*	F: AGGTACCTCTCAGGCCGATT R: GCTGCTTGACACCCAATGTG	133	94.13%	0.9944	−3.4710
*RPS5*	*Ribosomal protein S5*	F: GGTACGCACACAAGCGATTC R: CGCGACCAATCCTAGTGGAA	228	96.01%	0.9996	−3.4214
*RPL10*	*Ribosomal Protein L10*	F: CTGGAACTTGATGGTGGCCT R: GACGCTGAAGCCCTCAAAAA	219	91.67%	0.9993	−3.5392
*RPL13*	*Ribosomal Protein L13*	F: CTCCTGAGAGCGTCCTTTCC R: AAACGCTGCAATGTGAACCC	125	100.82%	0.9968	−3.3024
*RPL27*	*Ribosomal Protein L27*	F: ACGCGTGTGTTGAAACGTAG R: CCAAAGTCAAGCCCTTCGTG	145	92.58%	0.9998	−3.5142
*GAPDH*	*Glyceraldehyde-3-phosphate dehydrogenase*	F: AACTTGGTCCTCGGTGTATC R: CGTTGTTGACTTGACTGTCC	121	94.23%	0.9989	−3.4683
*AK*	*Arginine Kinase*	F: GATGACGGTGCAAGCGAAAT R: TCATCAGGCACGAGCTTACC	207	93.44%	0.9987	−3.4897

^a^ Amplicon length; ^b^ RT-qPCR efficiency determined using the standard curve method; ^c^ Regression coefficient calculated using the regression line of the standard curve.

**Table 2:** Rank order of candidate reference genes under different treatments in *Hyblaea puera*.

Conditions	Rank	ΔCt		BestKeeper		Normfinder		geNorm	
		Gene	Stability	Gene	Stability	Gene	Stability	Gene	Stability
Temperature	1	*RPL27*	1.26	*Beta*	0.46	*RPL10*	0.573	*RPL10*|*RPL27*	0.556
	2	*RPL10*	1.28	*28S*	0.463	*RPL27*	0.579		
	3	*28S*	1.39	*RPL27*	0.686	*28S*	0.865	*Beta*	0.986
	4	*Actin*	1.44	*RPL10*	0.76	*Beta*	0.923	*28S*	1.043
	5	*Beta*	1.45	*Actin*	0.785	*Actin*	0.940	*Actin*	1.112
	6	*AK*	1.49	*EF-1a*	0.999	*AK*	0.988	*EF-1a*	1.173
	7	*EF-1a*	1.53	*AK*	1.116	*EF-1a*	1.078	*AK*	1.235
	8	*RPL13*	1.64	*RPL13*	1.171	*RPL13*	1.350	*RPL13*	1.320
	9	*GAPDH*	1.95	*RPS5*	1.396	*GAPDH*	1.758	*GAPDH*	1.426
	10	*RPS5*	2.02	*GAPDH*	1.619	*RPS5*	1.848	*RPS5*	1.546
Larval instars	1	*RPL10*	1.70	*28S*	0.523	*RPL10*	0.779	*Actin*|*28S*	0.785
	2	*Actin*	1.87	*Actin*	0.582	*Actin*	1.172		
	3	*28S*	1.89	*EF-1a*	0.757	*28S*	1.279	*EF-1a*	0.894
	4	*EF-1a*	1.92	*RPL10*	1.267	*EF-1a*	1.327	*RPL10*	1.384
	5	*RPL13*	1.95	*RPS5*	1.285	*RPL13*	1.395	*RPL13*	1.624
	6	*RPL27*	2.08	*AK*	1.595	*RPL27*	1.494	*RPL27*	1.730
	7	*GAPDH*	2.14	*RPL27*	1.639	*GAPDH*	1.656	*GAPDH*	1.793
	8	*Beta*	2.26	*RPL13*	1.659	*Beta*	1.788	*Beta*	1.855
	9	*AK*	2.40	*Beta*	1.779	*AK*	1.894	*AK*	1.976
	10	*RPS5*	2.41	*GAPDH*	2.11	*RPS5*	2.080	*RPS5*	2.062
Adult sexes	1	*28S*	0.46	*EF-1a*	0.05	*AK*	0.125	*EF-1a*|*RPS5*	0.179
	2	*RPS5*	0.47	*RPS5*	0.112	*28S*	0.185		
	3	*AK*	0.47	*28S*	0.194	*RPS5*	0.234	*28S*	0.226
	4	*Beta*	0.52	*Beta*	0.226	*Beta*	0.324	*Beta*	0.253
	5	*RPL27*	0.54	*Actin*	0.296	*RPL27*	0.326	*AK*	0.301
	6	*EF-1a*	0.55	*AK*	0.331	*EF-1a*	0.420	*Actin*	0.343
	7	*Actin*	0.59	*RPL27*	0.500	*Actin*	0.435	*RPL27*	0.406
	8	*RPL10*	0.67	*GAPDH*	0.567	*RPL10*	0.581	*RPL10*	0.476
	9	*RPL13*	0.71	*RPL10*	0.704	*RPL13*	0.640	*RPL13*	0.516
	10	*GAPDH*	0.86	*RPL13*	0.776	*GAPDH*	0.781	*GAPDH*	0.585
Total developmental stages	1	*RPL10*	1.87	*Actin*	0.578	*RPL10*	0.551	*RPL10*|*RPL13*	1.005
	2	*EF-1a*	2.08	*28S*	0.985	*EF-1a*	1.135		
	3	*RPL13*	2.11	*EF-1a*	1.016	*RPL27*	1.220	*RPL27*	1.194
	4	*RPL27*	2.12	*RPS5*	1.144	*RPL13*	1.325	*GAPDH*	1.380
	5	*Actin*	2.20	*RPL10*	1.263	*Actin*	1.435	*AK*	1.679
	6	*RPS5*	2.43	*RPL27*	1.606	*AK*	1.836	*EF-1a*	1.855
	7	*GAPDH*	2.48	*AK*	1.794	*RPS5*	1.846	*Actin*	1.967
	8	*AK*	2.50	*RPL13*	1.945	*GAPDH*	1.914	*RPS5*	2.053
	9	*28S*	3.01	*Beta*	2.389	*Beta*	2.629	*28S*	2.223
	10	*Beta*	3.01	*GAPDH*	2.515	*28S*	2.666	*Beta*	2.381
Tissue types	1	*RPL10*	1.91	*EF-1a*	0.403	*AK*	0.264	*Beta*|*RPS5*	0.292
	2	*28S*	1.91	*28S*	0.419	*RPL10*	0.289		
	3	*RPL13*	1.95	*RPS5*	0.459	*RPL13*	0.289	*EF-1a*	0.663
	4	*RPS5*	1.96	*RPL10*	0.536	*28S*	0.367	*28S*	0.769
	5	*EF-1a*	1.99	*Beta*	0.595	*EF-1a*	0.409	*RPL10*	0.871
	6	*Beta*	2.06	*RPL13*	0.667	*RPS5*	0.806	*RPL13*	0.917
	7	*AK*	2.30	*AK*	1.104	*Beta*	1.012	*AK*	1.045
	8	*GAPDH*	2.38	*Actin*	1.172	*GAPDH*	1.720	*GAPDH*	1.156
	9	*Actin*	2.60	*GAPDH*	1.198	*Actin*	2.059	*Actin*	1.278
	10	*RPL27*	8.85	*RPL27*	6.764	*RPL27*	8.810	*RPL27*	2.792
All Conditions	1	*EF-1a*	2.16	*28S*	0.622	*EF-1a*	1.065	*EF-1a*|*RPS5*	1.235
	2	*RPL10*	2.25	*Actin*	0.838	*RPL10*	1.235		
	3	*Actin*	2.33	*RPS5*	1.101	*RPL13*	1.448	*Actin*	1.391
	4	*RPL13*	2.33	*EF-1a*	1.125	*Actin*	1.515	*28S*	1.504
	5	*RPS5*	2.45	*Beta*	1.498	*AK*	1.615	*RPL10*	1.824
	6	*AK*	2.52	*AK*	1.647	*RPS5*	1.715	*RPL13*	1.970
	7	*28S*	2.57	*RPL10*	1.813	*28S*	1.856	*AK*	2.077
	8	*Beta*	2.67	*RPL13*	1.869	*Beta*	1.916	*GAPDH*	2.163
	9	*GAPDH*	2.68	*GAPDH*	2.036	*GAPDH*	2.015	*Beta*	2.236
	10	*RPL27*	4.08	*RPL27*	2.523	*RPL27*	3.757	*RPL27*	2.605

**Table 3:** Recommended reference genes from RefFinder analysis for *Hyblaea puera* under various experimental conditions.

Conditions	Reference Gene	Conditions	Reference Gene
Tissue types	*RPL10*, *RPS5*	Adult sexes	*RPS5*, *EF-1a*
Temperature	*RPL27*, *RPL10*	Larval instars	*Actin*, *RPL10*
Total developmental stages	*RPL10*, *EF-1a*	All conditions	*EF-1a*, *Actin*

## Data Availability

The original data presented in this study are included in the article. Further inquiries can be directed to the corresponding author.

## Supplementary Materials

The following supporting information can be downloaded at: https://www.mdpi.com/article/10.3390/insects17060639/s1, Figure S1: Melting curve analysis of 10 candidate reference genes; Text S1: The nucleotide sequences of the PCR products of ten candidate reference genes from *Hyblaea puera*. Text S2: The nucleotide sequences of the longevity signaling pathway gene Lethal from *Hyblaea puera*.
