# Supplementary material for: Reference Genes for Expression Analyses by RT-qPCR in Hyblaea puera (Lepidoptera: Hyblaeidae)
Source: Insects. 2026 Jun 17;17(6):639. doi: 10.3390/insects17060639 (PMC13300253; doi:10.3390/insects17060639)
Supplement: Supplementary file 1 [file insects-17-00639-s001.zip › insects-4277001-supplementary.pdf]

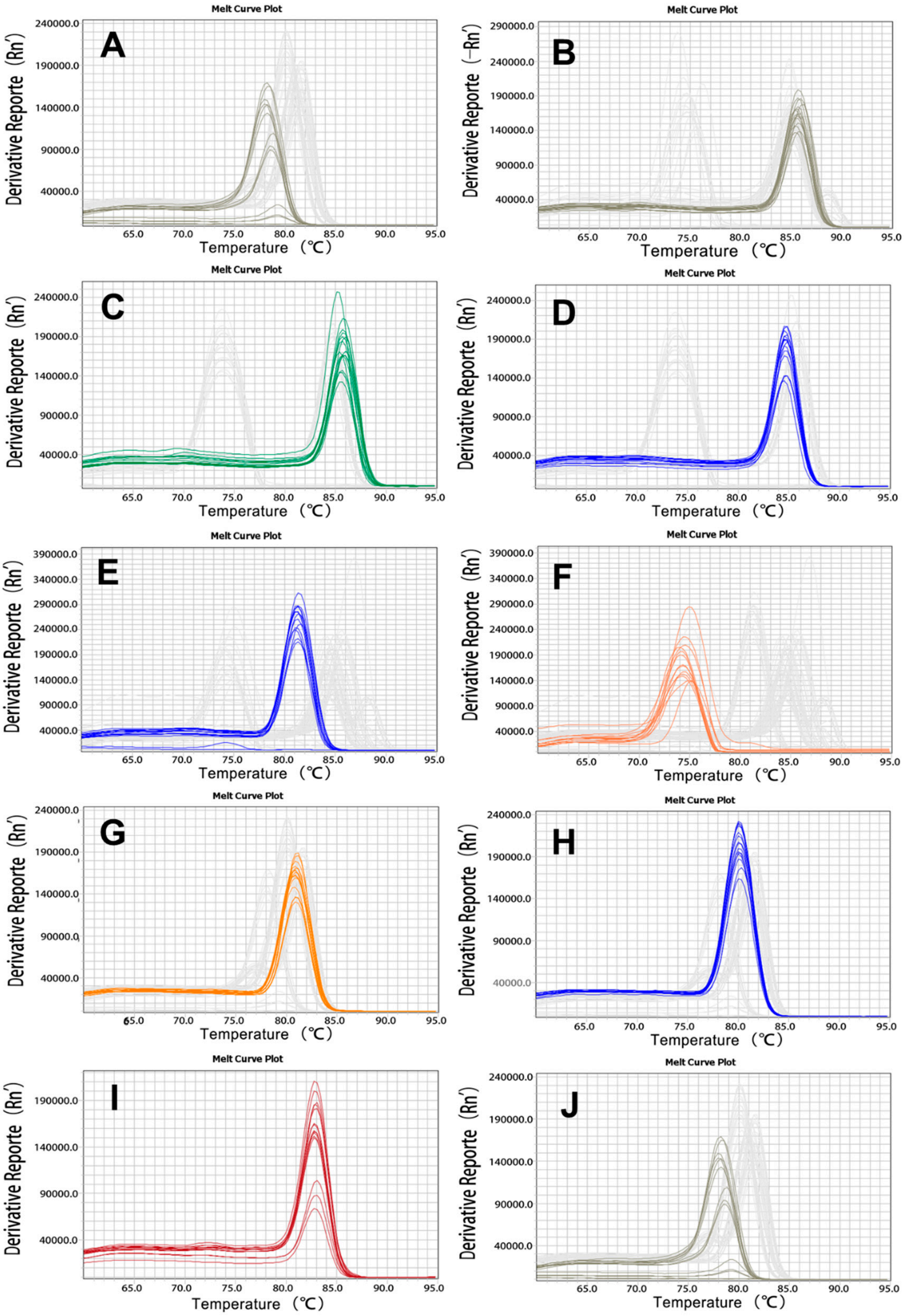

Figure S1. Melting curve analysis of 10 candidate reference genes.

A, *Actin*; B, *Beta-tubulin* (*Beta*); C, *28S ribosomal RNA* (*28S*); D, *Elongation factor-1α* (*EF-1a*); E, *Ribosomal protein S5* (*RPS5*); F, *Ribosomal Protein L10* (*RPL10*); G, *Ribosomal Protein L13* (*RPL13*); H, *Ribosomal Protein L27* (*RPL27*); I, *Glyceralde-hyde-3-phosphate dehydrogenase* (*GAPDH*); J, *Arginine Kinase* (*AK*).

Text S1. The nucleotide sequences of the PCR products of ten candidate reference genes from *Hyblaea puera*

Actin
TACTCTTTCACCACCACCGCTGAGAGGGAAATCGTCCGTGACATCAAGGAGA
AGCTGTGCTATGTCGCCCTCGACTTCGAGCAGGAGATGGCCACCGCTGCCGC
CTCCACCTCCCTCGAGAAGTCCTACGAGCTTCCCGACGGTCAGGTCATCACC
ATCGGTAACGAGAGGTTCCGTTGCC

Beta-tubulin (*Beta*)
ATGTCTGGTGTGACGACGTGCCTGCGCTTCCCGGGGCAGCTGAACGCGGACC
TGCGCAAGCTGGCGGTGAACATGGTTCCCTTCCCGCGGCTGCACTTCTTCATG
CCCGGGTTCGCCCCACTGACGTCGCGCGGCAGCCAGCAGTATCGCGCGCTCA
CCGTGCCAGAGTTGACGCAGCAGATGTTCGACGCCAAGAACATGATGGC

28S ribosomal RNA (*28S*)
GGCCGTTGGACGGTATATTATGTGAATCGCGCACGCTTTTAGCGTCCGGCCCG
ACGCAAGGTTATGCATCCGTCGATTTCCTGCCCATGTGCGGACGTTGGCGCTG
CGTGCCTGTTGTCGCTGCCGTGCAGTCTCGGACTGTGTGCGTCTCTGTCTGCG
ATGTTTCCGTTTCGGGCAC

Elongation factor-1α (*EF-1a*)
AGGTACCTCTCAGGCCGATTGCGCCGTGCTGATCGTCGCCGCCGGTACTGGT
GAGTTCGAGGCTGGTATCTCCAAGAACGGCCCAGACCCGTGGAGCACGCTCT
GCTCGCTTTCACATTGGGTGTCAAGCAGC

Ribosomal protein S5 (*RPS5*)
GGTACGCACACAAGCGATTCCGCAAAGCGCAATGCCCCATCGTTGAACGTCT
CACCAACTCCTTGATGATGCACGGACGTAACAATGGCAAGAAATTGATGGCT
GTTCGAATTGTTAAGCACGCCTTTGAAATTATCCACTTGCTAACTGGTGAGAA
CCCACTCCAGGTTCTTGTGACTGCCATCATTAACTCAGGACCCCGTGAAGATT
CCACTAGGATTGGTCGCG

Ribosomal Protein L10 (*RPL10*)
CTGGAACTTGATGGTGGCCTTGACCTCATCGATCTTCTGTGTCATGGACTCCT
GGTGGGAGAGGAGACCAGGGAACTTACCGGCCTTGTTCAGACCGGGACCGA
GGAGACGTGGGATCTGTTTGATCAAGGACTCTGATGCGAGGAAAGCATCATA
TTTCTTAGCTAGTTTCTTGACAAGTTTCTTGTTCTTGTTCAATTTTTTGAGGGCT
TCAGCGTC

Ribosomal Protein L13 (*RPL13*)

CTCCTGAGAGCGTCCTTTCCACGCTCAGTCTTGTGTGGGATCATTCCTCTGAC
GGTCTTCCACAGAATCTTGGAAGGAGCTCTGAAGTGGAAAGGTCCACGGGCT
GGGTTCACATTGCAGCGTTT

Ribosomal Protein L27 (*RPL27*)
ACGCGTGTGTTGAAACGTAGCTTCTTGCGTTTTGCGGGGTCTTTCAGGTCCTTT
GCACTGAACTTATCAAAGCTGAAGTCTACTGAGTAACGGGTGGGCATCAAGT
GGTTGTAGTTGACGACCTTCACGAAGGGCTTGACTTTGG

Glyceralde-hyde-3-phosphate dehydrogenase (*GAPDH*)
AACTTGGTCCTCGGTGTATCCAAGGATGCCCTTGAGGGGACCTTCAGCGGCC
TCTTTGACCTTCTGCTTGATGGCATCGTAGCTGGCGGGCTTGCCCAGGCGGAC
AGTCAAGTCAACAACG

Arginine Kinase (*AK*)
GATGACGGTGCAAGCGAAATTATTAAAAACTTTAATATTAGGAGCCCCAGCA
TCTGGGAAAGGTACCATATCTTCGCGTATAGTGAAGAGGTATAATATCGAGC
ATGTATCAAGTGGTGATAAGTTGCGTGACCATATTCAGAAGAAAACTGAGCT
AGGAAAAGAAGTGCAGAAGTATTTGGACGAAGGTAAGCTCGTGCCTGATGA

Text S2. The nucleotide sequences of the longevity signaling pathway gene *Lethal* from *Hyblaea puera*
TCGTTCGTTCTTCAATGCTGCAGCGACCCTAGGAGCGACCACGGTCAGCACA
CCATCAGATGATAACTTAGACTCGACTGTTTCAGGGTTGCAACCTTCGGGAA
GGGCGTAGCGGCGGGTGAACTGCCTCGAGATGTATCCGTGCTCGTCTTTCTTC
TCCTCGTGCTTGCCTTCGACGATGACGTACCCATCAGCGGTCTTCACACTGAT
TTCCTCTGGGGCGAAATGCTGCACATCCAGATTGACTTGGAACTTTTCCTTAT
CGGACTTGATAGTGGATCCGATGTCTCTCGCAGCAGCGGCCATCTGACGCCA
GGGCCTGTAGTAGTCCCGGGACATCATCGGAGCCACGGCAGCGGTGAGCAA
GTCGTCCGGAGTCAGCGCCAGACCGAAGTCCTGTTCCAACAGGCGATGGGG
GTGTTCGTAGCCAACGATGAACGGTAGCAGAGACATCTTCCTTTTTCTTCTTA
GCTTAATATTCGAGTTGTGAGATCGCAG
